# Supplementary material for: The effects of sociodemographic factors on help-seeking for depression: Based on the 2017–2020 Korean Community Health Survey
Source: PLoS One. 2023 Jan 19;18(1):e0280642. doi: 10.1371/journal.pone.0280642 (PMC9851551; doi:10.1371/journal.pone.0280642)
Supplement: Attachment — Submitted filename: Review comments_PONE-D-22-18284.pdf [file pone.0280642.s001.pdf]

Review comments on the manuscript PONE-D-22-18284

Dear authors,

Thank you for your research article and your work in this important scientific field. The article is very concise (maybe too conscience). Some changes should be made to improve readability and scientific transparency before publication.

Yours sincerely and all the best,

Your anonymous reviewer ☺

#	Review Comment	Lines
1	Corresponding mail: If possible a more professional mail address than jayun5959@gmail.com	16
2	The term "*who did not receive national basic security*" is no understandable for me (as a non-Korean reader?). Clarify please	40-41
3	*were more likely not to* → confusing statement. Better: *were less likely to …*	41
4	*with depressed individuals* → stigmatising. Please use "people first" language in the whole article (e.g., individuals with depressive symptoms)	44
5	Keyword: "psychiatric treatment" doesn't fit in my opinion, because …*"Help-seeking behavior was measured as receiving counseling from professionals (medical institutions, professional counseling institutions, public health centers, etc.)"* → professional treatment might be better	48<br>Citation taken from lines: 35-37
6	*People with depression tend not to seek help because they believe they should solve depressive symptoms on their own without external help* → This doesn't seem a fitting citation because (16) is for youth and adolescents, however here the article authors generalise it for "people". Furthermore, "wanting to solve issues by oneself" is not something that is so bad, yet here the authors state it in the context with stigmatising attitudes. Why?	77-79
7	*Especially, depression in the productive population causes more social burden* → no citation? This seems necessary to me for such a statement	84
8	Concerning Stigma: Too short paragraph with little reflection of different types of stigmatising attitudes and how they might impact the help-seeking process of people with depressive symptoms. I suggest that the authors reflect a little more on stigma. Such as "internalised", "anticipated" and "help-seeking" sigma are possible search word I suggest looking up in this context.<br><br>Also, why is it discussed in the theory if it was not measured and therefore not included in the results?	75-79

9	*using the standardized method of the Korean Community Health Survey* → confusing sentence, since the Survey is not a standardised method…? Is it a survey that is repeatedly issued ever so often? Clarify please.	88
10	*Patients with unemployment were excluded from the study.* → Why? These are an especially vulnerable group (also for depression) and concerning stigma/help-seeking and financial barriers you wrote about above, they seem to be of special interest? Please clarify the rational.	103-104
11	*Measures* chapter should be revised extensively!<br>There are hardly any citations. No item examples, scale (Likert? What range…?), reliability scores (Cornbach's Alpha for example). Are any composite cores calculated.<br>Also, was help-seeking behaviour assessed as a Yes/No answer? Specifically for seeking help because of depressive symptoms or in general? This chapter is lacking.<br><br>Rational is missing for:<br>• Why were ages grouped like this?<br>• Why was income grouped like this (is it usually done like this in other articles)?<br>• Why was occupation grouped like this? – also why not unemployment (still wondering about this).	111-134
12	Nine points on the Patient Health Questionnaire-9 (PHQ-9). Why 9? It is just stated that you reviewed the literature, yet no argument is given. Is it the most common cut-off in Korean literature, what does it signify? I myself know of 5, 8 and 10 as usual cut-offs.	130-131
13	*Subjective stress* → please explain whether this has been done elsewhere or not? Is it original?	131
14	*Furthermore, we conducted a factor analysis on the PHQ-9 to explore which depressive symptoms had a greater effect on help-seeking.* → This information belongs in the *Statistic Analysis* chapter	133-134
15	*Statistical Analysis* chapter is also lacking in information. Which test were used exactly? Was checked for test prerequisites? I suggest building the sentences as follows: To analyse if … we conducted … tests after checking for … prerequisites (or in a similar manner).<br><br>Remember. I have to understand exactly what you did and why so that (especially since you allow for data sharing) I could replicate your findings exactly after having read your article.	137-147
16	Principal component analysis with direct oblimin rotation (δ=0) was used to extract the main factors with eigenvalues >1.0. → This is a good example for information that sadly doesn't tell me why you did this?	146-147
17	Formatting: Use "." for big numbers to approve readability. Sometimes you do this, sometimes not? Ex., this is annoying to read: *Of the 561,654 people, 16,390 received more than nine points on the PHQ-9, 25,790 felt sad or hopeless*	152-153

18	Table 1 is a good overview. However, please format (e.g., capital letters someplace, other places small letter) and you must ad a "note" under the table to explain it. Tests used, "*"-meanings etc. (refer to reporting standards). Tables should be able to "stand by themselves" with no further information needed from the text (which is lacking due to the brief measures section anyhow).	table 1
19	*A previous study (Jeon et al., 2020) using the BDI-II suggested that there were two latent factors of depression, and we also expected that the PHQ-9 would have two factors. As expected, two factors were extracted: one factor was related to somatic affective symptoms (items 1 to 5), and the other was a cognitive factor related to depression (items 6 to 9).* → This belongs in the theory and methods chapter (at the very least the method chapter). Now I understand why you write this in the statistical analysis chapter. Please revise. (see comment #16)	165-169
20	Table 2 title is missing – please amend. The "note" information is lacking. See comment 18. Also, the note that is written is confusing, because no major loadings are shown in "bold face"?	table 2
21	Results: Generally, why do the authors report who is less likely to seek help instead of saying who is more likely to do so. Is more positive and maybe also more helpful. At least it should always be juxtaposed to the reference groups. Ex.: *Males were more likely 177 not to seek help for depression than females (aOR=1.31; 95% CI=1.157-1.487). Individuals aged 45-64 or 75 or more were more likely not to seek help for depression than those aged 19-44 (45-64 aged aOR=1.19; 95% CI=1.022-1.389; 75 or more aOR=1.45; 95% CI=1.059-1.973).* → Possibly it is also a language issue: "more likely not to seek hep" = "less likely to seek help"	Ex.: 177-180
22	Table 3 better than 1 an 2. However, why are some aOR bold faced and others are not? The heading should also be more descriptive so that the reader knows straight away what they are looking at.<br>O.R. should be aOR, otherwise not correct abbreviation → these mistakes should be revised before publication!	table 3
23	*For example, in the case of males, masculine social norms such as self-reliance and emotional control can make it difficult for males to seek help when they acknowledge their depression [27].* → The citation might not be chosen so well, because as I understand it refers to more "Western" masculinity norms; are there better sources for Korean men/masculinity norms? The same goes for the citation #28	194-198
24	The issue of socioeconomic status as a "fundamental cause" for health inequality should be discussed. Especially in light of these findings! The authors have great basis for building the SES-Index with the questions they assessed. It is not necessary, however, it might add positively to the regression model if the SES-Index is used. Irrespectively if done so or	discussion

	not, it should be discussed, especially as it was “teased at” as an obstacle in line 75)	
25	The discussion is lacking and too short. I would expect more detail on the design and measurements and more detailed reflexion on the current research and how your findings fit into this “bigger picture”. Must be enhanced before publication.	discussion
26	*Generally:* Some small English mistakes or uncommon use of words or interesting sentence structure – please revise for better readability. (ex.: Since approximately 230,000 participants completed the KCHS annually, a total of 915,089 participants completed it over four years, line 102-103)	
27	*Yes - all data are fully available without restriction* → How exactly? For non Korean readers it is impossible to understand the website. Is there a more internationally friendly option?	data availability
