# Supplementary material for: The effects of sociodemographic factors on help-seeking for depression: Based on the 2017–2020 Korean Community Health Survey
Source: PLoS One. 2023 Jan 19;18(1):e0280642. doi: 10.1371/journal.pone.0280642 (PMC9851551; doi:10.1371/journal.pone.0280642)
Supplement: Attachment — Submitted filename: 221110_Review comments_PONE-D-22-18284_R1.pdf [file pone.0280642.s003.pdf]

Review comments on the manuscript PONE-D-22-18284R1 (revised)

Dear authors,

Thank you for incorporating some of my criticism into your work. I have reread the article and the points you changed and answer in detail in the table below. I have crossed through the points that I find have been satisfactorily changed. Some points should however still be considered (my new comments in red).

I know you are not native English speakers so I kindly suggest you let a professional proof reader correct your draft.

Yours sincerely and all the best in your work,

Your anonymous reviewer

#	Review Comment	Lines
~~1~~	~~Corresponding mail: If possible a more professional mail address than jayun5959@gmail.com~~	~~16~~
2	The term "*who did not receive national basic security*" is not understandable for me (as a non-Korean reader?). Clarify please<br><br>➔ Thank you for clarifying in the measures section.<br>➔ Please write in the abstract more precisely. For example: *"(…) that males, aged 45-64 years and those more than 75 years, who did not have Medical Aid program, and less educated people were less likely to seek help for depression."* → This sentence is too ambiguous. This is only one example that the authors find it difficult to use clear English. The article would benefit from a professional proof reader.	40-41 (now lines 131 and following)
~~3~~	~~*were more likely not to* → confusing statement. Better: *were less likely to …*~~	~~41~~
~~4~~	~~*with depressed individuals* → stigmatising. Please use "people first" language in the whole article (e.g., individuals with depressive symptoms)~~	~~44~~
~~5~~	~~Keyword: "psychiatric treatment" doesn't fit in my opinion, because …"*Help-seeking behavior was measured as receiving counseling from professionals (medical institutions, professional counseling institutions, public health centers, etc.)"* → professional treatment might be better~~	~~48~~<br>~~Citation taken from lines: 35-37~~
~~6~~	~~*People with depression tend not to seek help because they believe they should solve depressive symptoms on their own without external help* → This doesn't seem a fitting citation because (16) is for youth and adolescents, however here the article authors generalise it for "people". Furthermore, "wanting to solve issues by oneself" is not something that is so bad, yet here the authors state it in the context with stigmatising attitudes. Why?~~	~~77-79~~

~~7~~	~~*Especially, depression in the productive population causes more social burden* → no citation? This seems necessary to me for such a statement~~	~~84~~
~~8~~	~~Concerning Stigma: Too short paragraph with little reflection of different types of stigmatising attitudes and how they might impact the help-seeking process of people with depressive symptoms. I suggest that the authors reflect a little more on stigma. Such as "internalised", "anticipated" and "help-seeking" sigma are possible search word I suggest looking up in this context.~~<br><br>~~Also, why is it discussed in the theory if it was not measured and therefore not included in the results?~~	~~75-79~~
~~9~~	~~*using the standardized method of the Korean Community Health Survey* → confusing sentence, since the Survey is not a standardised method…? Is it a survey that is repeatedly issued ever so often? Clarify please.~~	~~88~~
10	*Patients with unemployment were excluded from the study.*<br>→ Why? These are an especially vulnerable group (also for depression) and concerning stigma/help-seeking and financial barriers you wrote about above, they seem to be of special interest? Please clarify the rational.<br><br>➔ Although I appreciate the changes made, I still find the exclusion to be problematic. It would, in my opinion, be more prudent to keep people with no current employment in the sample and categorise them as the 5. category in "occupation". In the way the rational is presented here, there is an implicit discrimination in the study against people with no current employment. You argue "Since various sociodemographic factors are related to help-seeking behavior and depression, it is difficult to generalize results of research to all population groups in the community"(ll. 84-86) and then exclude a part of the population to analyse the "productive population", making the findings in this study less generalizable.	103-104
11	*Measures* chapter should be revised extensively!<br>There are hardly any citations. No item examples, scale (Likert? What range…?), reliability scores (Cornbach's Alpha for example). Are any composite cores calculated.<br>Also, was help-seeking behaviour assessed as a Yes/No answer? Specifically for seeking help because of depressive symptoms or in general? This chapter is lacking.<br><br>Rational is missing for:<br>• Why were ages grouped like this?<br>• Why was income grouped like this (is it usually done like this in other articles)?<br>• Why was occupation grouped like this? – also why not unemployment (still wondering about this).	111-134

	➔ All in all this is a lot better! However, reliability scores are still missing for PHQ-9 and subjective stress (why?)	
~~12~~	~~Nine points on the Patient Health Questionnaire-9 (PHQ-9). Why 9? It is just stated that you reviewed the literature, yet no argument is given. Is it the most common cut-off in Korean literature, what does it signify? I myself know of 5, 8 and 10 as usual cut-offs.~~	~~130-131~~
13	*Subjective stress* → please explain whether this has been done elsewhere or not? Is it original?<br><br>➔ This is still not satisfactorily explained (or maybe the KCHS wrote the items themselves without reference to previous studies?)	131
~~14~~	~~*Furthermore, we conducted a factor analysis on the PHQ-9 to explore which depressive symptoms had a greater effect on help-seeking.* → This information belongs in the *Statistic Analysis* chapter~~	~~133-134~~
~~15~~	~~*Statistical Analysis* chapter is also lacking in information. Which test were used exactly? Was checked for test prerequisites? I suggest building the sentences as follows: To analyse if … we conducted … tests after checking for … prerequisites (or in a similar manner).~~<br><br>~~Remember. I have to understand exactly what you did and why so that (especially since you allow for data sharing) I could replicate your findings exactly after having read your article.~~	~~137-147~~
~~16~~	~~Principal component analysis with direct oblimin rotation (δ=0) was used to extract the main factors with eigenvalues >1.0. → This is a good example for information that sadly doesn't tell me why you did this?~~	~~146-147~~
17	Formatting: Use "." for big numbers to approve readability. Sometimes you do this, sometimes not? Ex., this is annoying to read: *Of the 561,654 people, 16,390 received more than nine points on the PHQ-9, 25,790 felt sad or hopeless*<br><br>➔ The changes are helpful, but why not use . instead of , to write big numbers?	152-153
~~18~~	~~Table 1 is a good overview. However, please format (e.g., capital letters someplace, other places small letter) and you must ad a "note" under the table to explain it. Tests used, "*"-meanings etc. (refer to reporting standards). Tables should be able to "stand by themselves" with no further information needed from the text (which is lacking due to the brief measures section anyhow).~~	~~table 1~~
~~19~~	~~*A previous study (Jeon et al., 2020) using the BDI-II suggested that there were two latent factors of depression, and we also expected that the PHQ-9 would have two factors. As expected, two factors were extracted: one factor was related to somatic affective symptoms (items 1 to 5), and the other was a cognitive factor related to depression (items 6 to 9).* → This belongs in the theory and methods chapter (at the very~~	~~165-169~~

	~~least the method chapter). Now I understand why you write this in the statistical analysis chapter. Please revise. (see comment #16)~~	
20	Table 2 title is missing – please amend. The "note" information is lacking. See comment 18. Also, the note that is written is confusing, because no major loadings are shown in "bold face"?<br><br>➔ Table 2 title is still missing. These superficial things should be done with care! Please amend.	table 2
21	Results: Generally, why do the authors report who is less likely to seek help instead of saying who is more likely to do so. Is more positive and maybe also more helpful. At least it should always be juxtaposed to the reference groups. Ex.: *Males were more likely 177 not to seek help for depression than females (aOR=1.31; 95% CI=1.157-1.487). Individuals aged 45-64 or 75 or more were more likely not to seek help for depression than those aged 19-44 (45-64 aged aOR=1.19; 95% CI=1.022-1.389; 75 or more aOR=1.45; 95% CI=1.059-1.973).* → Possibly it is also a language issue: "more likely not to seek hep" = "less likely to seek help"<br><br>➔ Generally in multiple regressions, it is more usual to report the findings in reference to the reference group. I didn't notice this before due to the double negative, but for example I suggest writing the results as follows: "It was found X were more likely to seek help for depression than Y" (and Y is the reference group)<br>➔ Also, there is a confusing presentation of the multiple regression results that need to be simplified. You code "help-seeking" as 0 and "non-help seeking" as 1. That would mean that those groups with aOR>1 were more likely not to seek help compared to the reference group and this leads to confusing double negatives in the results section: e.g.: "Individuals who did not have Medical Aid program were less likely to seek help for depression than individuals with Medical Aid program" (l. 205). It would be a lot easier for the reader if a) the help-seeking outcome were coded as 0=no help and 1=help seeking and the results section written with simple "X was more likely then Y to seek help" or "X was less likely then Y to seek help" (Y being the reference category).<br>➔ I could only find the categorisation as 0=help seeking and 1=no help-seeking in the note of table 3, which I first didn't read and then was confused to the result interpretation. It should also be transparently written so in the paragraph "statistical analysis".	Ex.: 177-180
~~22~~	~~Table 3 better than 1 an 2. However, why are some aOR bold faced and others are not? The heading should also be more descriptive so that the reader knows straight away what they are looking at.~~	~~table 3~~

	~~O.R. should be aOR, otherwise not correct abbreviation → these mistakes should be revised before publication!~~	
~~23~~	~~*For example, in the case of males, masculine social norms such as self-reliance and emotional control can make it difficult for males to seek help when they acknowledge their depression [27].* → The citation might not be chosen so well, because as I understand it refers to more "Western" masculinity norms; are there better sources for Korean men/masculinity norms? The same goes for the citation #28~~	~~194-198~~
~~24~~	~~The issue of socioeconomic status as a "fundamental cause" for health inequality should be discussed. Especially in light of these findings! The authors have great basis for building the SES-Index with the questions they assessed. It is not necessary, however, it might add positively to the regression model if the SES-Index is used. Irrespectively if done so or not, it should be discussed, especially as it was "teased at" as an obstacle in line 75)~~	~~discussion~~
~~25~~	~~The discussion is lacking and too short. I would expect more detail on the design and measurements and more detailed reflexion on the current research and how your findings fit into this "bigger picture". Must be enhanced before publication.~~	~~discussion~~
26	*Generally:* Some small English mistakes or uncommon use of words or interesting sentence structure – please revise for better readability. (ex.: Since approximately 230,000 participants completed the KCHS annually, a total of 915,089 participants completed it over four years, line 102-103)<br><br>→ English is still lacking in clarity. And some sentences were not easy to understand.	
~~27~~	~~*Yes - all data are fully available without restriction* → How exactly? For non Korean readers it is impossible to understand the website. Is there a more internationally friendly option?~~	~~data availability~~
