# Supplementary material for: The effects of sociodemographic factors on help-seeking for depression: Based on the 2017–2020 Korean Community Health Survey
Source: PLoS One. 2023 Jan 19;18(1):e0280642. doi: 10.1371/journal.pone.0280642 (PMC9851551; doi:10.1371/journal.pone.0280642)
Supplement: Attachment — Submitted filename: Answer_to_Reviewer_2nd_1229.docx [file pone.0280642.s004.docx]

**ANSWERS TO REVIEWERS**

We thank the reviewer for your thoughtful suggestions and insights. The manuscript has benefited from these insightful suggestions. The manuscript has been rechecked and the necessary changes have been made in accordance with the reviewers’ suggestions. The responses to all comments have been prepared and attached herewith.

1. Please write in the abstract more precisely. For example: “(…) that males, aged 45-64 years and those more than 75 years, who did not have Medical Aid program, and less educated people were less likely to seek help for depression.” → This sentence is too ambiguous. This is only one example that the authors find it difficult to use clear English. The article would benefit from a professional proof reader.

- **Thank you for your advice. We have revised the abstract accordingly. Our manuscript has also been proofread thoroughly. (Line 40-44)**
- **Male participants (adjusted odds ratio [aOR]=1.31, 95% confidence interval [CI]=1.157–1.487), those aged 45–64 years (aOR=1.192, 95% CI=1.022–1.389) and more than 75 years (aOR=1.446, 95% CI=1.059–1.973), those not having a Medical Aid program (aOR=1.750, 95% CI=1.375–2.226), and those having low educational levels (aOR=.896, 95% CI=.830–.968) were less likely to seek professional help for depression.**

2. Although I appreciate the changes made, I still find the exclusion to be problematic. It would, in my opinion, be more prudent to keep people with no current employment in the sample and categorise them as the 5. category in “occupation”. In the way the rational is presented here, there is an implicit discrimination in the study against people with no current employment. You argue “Since various sociodemographic factors are related to help-seeking behavior and depression, it is difficult to generalize results of research to all population groups in the community”(ll. 84-86) and then exclude a part of the population to analyse the “productive population”, making the findings in this study less generalizable.

- **Thank you for your comment. Previous studies on help-seeking behavior have focused on vulnerable groups such as older adults and individuals with low socioeconomic status. Therefore, our study aimed to conduct an analysis targeting the working population to differentiate from the existing research topics. In addition, being unemployed can be a reason or a consequence of depression. However, as you indicated, if we had controlled for this factor in the analysis, it may have been more representative. We have added these points in the limitations section. (Lines 269-271)**
- **Second, this study targeted employed individuals alone; therefore, bias cannot be ruled out, and the results of this study cannot represent the entire community population.**

3. All in all this is a lot better! However, reliability scores are still missing for PHQ-9 and subjective stress (why?)

- **Thank you for your comment. We have analyzed the reliability score and added it. (Line 155)**
- **Cronbach's alpha for the PHQ-9 was 0.802 in the present study.**

4. This is still not satisfactorily explained (or maybe the KCHS wrote the items themselves without reference to previous studies?)

- **The KCHS survey gathered information through face-to-face interactions between trained interviewers and respondents. Data on subjective stress were also obtained through interviews using one simple question. We have added these points in the limitations section. (Lines 274-277)**
- **although a trained interviewer conducted the interview to measure depression and subjective stress, the participants were not clinically diagnosed with depression by a mental health professional. Moreover, subjective stress was measured by one simple question rather than a validated tool.**

5. The changes are helpful, but why not use . instead of , to write big numbers?

- **Thank you for your comment. In the US, UK, and China, a comma is placed after every 3 digit positions for numbers larger than 999. We have followed the guidelines of this journal.**

6. Table 2 title is still missing. These superficial things should be done with care! Please amend.

- **Thank you for your comment. We have added the table title as per your advice. (Line 197)**
- **Table2. The factor analyses of the PHQ-9 in the sample at study entry (N=915,089)**

7. Generally in multiple regressions, it is more usual to report the findings in reference to the reference group. I didn’t notice this before due to the double negative, but for example I suggest writing the results as follows: “It was found X were more likely to seek help for depression than Y” (and Y is the reference group)

Also, there is a confusing presentation of the multiple regression results that need to be simplified. You code “help-seeking” as 0 and “non-help seeking” as 1. That would mean that those groups with aOR>1 were more likely not to seek help compared to the reference group and this leads to confusing double negatives in the results section: e.g.: “Individuals who did not have Medical Aid program were less likely to seek help for depression than individuals with Medical Aid program” (l. 205). It would be a lot easier for the reader if a) the help-seeking outcome were coded as 0=no help and 1=help seeking and the results section written with simple “X was more likely then Y to seek help” or “X was less likely then Y to seek help” (Y being the reference category).

I could only find the categorisation as 0=help seeking and 1=no help-seeking in the note of table 3, which I first didn’t read and then was confused to the result interpretation. It should also be transparently written so in the paragraph “statistical analysis”.

- **Thank you for your comment. Our research goal was to identify factors related to help-seeking behavior to guide appropriate interventions. To emphasize the subject of this study, no help-seeking behavior was coded as '1' and analyses were performed. Based on your suggestion, we have added an explanation about the coding in the Method section to help readers understand better. (Lines 165-166)**
- **Help-seeking behavior was coded as 0, which stands for help-seeking behavior and 1, stands for no help-seeking behavior.**

8. English is still lacking in clarity. And some sentences were not easy to understand.

- **Thank you for your comment. The entire manuscript has been proofread thoroughly.**
